# The Vermiform Appendix and Its Diseases

**Published:** 1905-07

**Authors:** 


### BOOK REVIEWS.

THE VERMIFORM APPENDIX AND ITS DISEASES. By HOWARD A. KELLY, A.M., M.D., Professor of Gynecology in the Johns Hopkins University, Baltimore, and E. Hurdon, M.D., Assistant in Gynecology in the Johns Hopkins University. Royal octavo, pp. xx.-887. Illustrated with 399 original illustrations, some in colors, and 3 lithographic plates. Philadelphia and London: W. B. Saunders & Company. 1905. (Price: cloth, $10 net; sheep or half morocco, $11 net.)

This comprehensive and elaborate treatise is prepared by a professor and assistant professor of gynecology. The former has declared from the house-tops that his specialty is "passing," but this book does not confirm his own judgment. It seems to condemn him out of his own mouth, so to speak, for it is a work on a surgical disease written by gynecologists.

In dealing with the anatomy of the vermiform appendix the authors begin with the fetus and study its development from the end of the first month of intrauterine life until birth. The conclusion reached by this investigation is that the appendix does not maintain the relative development in the adult reached in the fetus, and it is regarded, therefore, as the distal portion of the original cecum. This would seem to dispose of many theories as to the purpose, present or obsolete, of this treacherous organ.

One of the most interesting phases of this treatise is the relation of foreign bodies or concretions to the disease. Formerly it was the general belief of the profession, as well as the laity, that disease of the appendix was caused in general by "grape seeds" and other foreign elements that made their way into the lumen of this attachment; now it is admitted that only in particular cases does this accident happen. Numerous examples of this complication are given herein, and a group of illustrations are presented delineating the part occasionally played by concretions and other foreign substances in the etiology of appendicitis.

The clinical features of this work are of special value, exactness of detail being a marked characteristic of their presentation. Perhaps, too, another principal point of its importance is the profuseness of its illustrations. Not a single anatomical, pathological, or surgical item has been omitted in the art department, and the quality of all classes of illustration has never been excelled in a medical work. The book lacks a general index, otherwise it is beyond criticism.

